# A New Metasurface Superstrate Structure for Antenna Performance Enhancement

**DOI:** 10.3390/ma6083226

**Published:** 2013-07-31

**Authors:** Mohammad Tariqul Islam, Mohammad Habib Ullah, Mandeep Jit Singh, Mohammad Rashed Iqbal Faruque

**Affiliations:** 1 Institute of Space Science (ANGKASA), Universiti Kebangsaan Malaysia, Bangi, Selangor 43600, Malaysia; E-Mails: habib_ctg@yahoo.com (M.H.U.); mandeep@eng.ukm.my (M.J.S.); rashedgen@yahoo.com (M.R.I.F.); 2 Department of Electrical, Electronic and System Engineering, Faculty of Engineering and Built Environment, Universiti Kebangsaan Malaysia, Bangi, Selangor 43600, Malaysia

**Keywords:** metasurface superstrate (MSS), ceramic filled bioplastic, dual band patch antenna, bandwidth and gain enhancement

## Abstract

A new metasurface superstrate structure (MSS)-loaded dual band microstrip line-fed small patch antenna is presented in this paper. The proposed antenna was designed on a ceramic-filled bioplastic sandwich substrate with a high dielectric constant. The proposed 7 × 6 element, square-shaped, single-sided MSS significantly improved the bandwidth and gain of the proposed antenna. The proposed MSS incorporated a slotted patch antenna that effectively increased the measured operating bandwidth from 13.3% to 18.8% and from 14.8% to 23.2% in the lower and upper bands, respectively. Moreover, the average gain of the proposed MSS-based antenna was enhanced from 2.12 dBi to 3.02 dBi in the lower band and from 4.10 dBi to 5.28 dBi in the upper band compared to the patch antenna alone. In addition to the bandwidth and gain improvements, more directive radiation characteristics were also observed from the MSS antenna compared to the patch itself. The effects of the MSS elements and the ground plane length on the reflection coefficient of the antenna were analyzed and optimized. The overall performance makes the proposed antenna appropriate for RFID and WLAN applications.

## 1. Introduction

Currently, in response to the increasing price of crude oil, environmental concerns about fossil resource depletion, greenhouse gas emissions, and pollution with durable plastics are significantly increasing. Eco-friendly bio-based plastic materials and composites have become more prevalent from hi-tech device producing market players [1]. The continuously increasing demand for bio-based plastics in microwave circuitry applications is further intensified because these materials are now being used for durable solutions. In particular, the use of bioplastics in disposable applications, such as packaging and catering items (crockery, cutlery, pots, bowls, and straws), has been widespread for decades due to their biodegradability [2]. However, bioplastics have remained unpopular for long-term applications, such as microwave circuitry and electronic devices. This shortcoming has been mitigated by using bio-polymer composites in their durable plastic form. One important concern is the use of plastic materials that have suitable dielectric properties for the intended application. For instance, it is necessary to use high dielectric constant substrates for miniaturized printed circuit board (PCB) devices. Miniaturization of the printed patch antenna is a prime example showcasing the use of high permittivity dielectric substrates. However, the impedance bandwidth of the patch antenna decreases with the incorporation of high dielectric constant substrates. Several researchers have studied printed microstrip patch antennas on sandwich/stacked/multilayer substrates. Microstrip patch antennas are physically robust and can easily integrate with microwave circuitry due to their simple planar characteristics [3]. However, microstrip patch antennas have some shortcomings compared to non-planar antennas, such as their narrow bandwidth, low gain and radiation efficiency. These drawbacks limit the use of microstrip patch antennas for a wide range of applications. To make the antenna suitable for multiple applications, such as RFID and WLAN, they are required to have a wide operating bandwidth and high gain [4]. Artificial materials have become popular for use in antenna design and have resulted in performance improvements over the past several years. Metamaterials (MTM) [5], artificial magnetic conductors (AMC) [6,7], electromagnetic band gap structures (EBG) [8,9,10], high impedance electromagnetic surfaces [6], photonic band gap (PBG) structures [11,12] and reactive impedance substrates (RIS) [13] are some of the more widely used artificial materials for antenna applications. The concept of artificial materials/metamaterials was initially introduced in 1967 [14], and numerous names and terminologies have been used for negative permittivity (*ε*) and permeability (*µ*) by different researchers, such as “left-handed metamaterial (LH-MTM)” media with a negative refractive index, “backward wave media”, and “double negative (DNG)” metamaterials [15].

Based on the revolutionary work of Enoch *et al.* concerning metamaterial-based broadside directivity enhancement [16], there has been an intensive research effort towards the development of metamaterials or metasurfaces [5,17]. Metasurfaces are uniformly distributed, 2D, periodic, planar elements that are equivalent to metamaterials that exhibit anomalous values in their constitutive parameters at certain frequencies. Holloway *et al.* illustrated a number of metasurfaces that can be used for circumventing the λ/2 resonator size limit [18]. One of the attractive mechanical advantages of metasurfaces is that they are easy to fabricate and occupy a smaller amount of space compared to other 3D metamaterial structures. According to Snell’s law of refraction, θ2 = arcsin(n1sinθ1/n2), when n1 ~ 0 and n2 = 1, θ2 will be ~0 for all θ1. This is equivalent to the electromagnetic wave that originates from a principal source implanted inside a material block that has a near-zero refractive index. Therefore, the electromagnetic wave is intense at a slight solid angle around the aperture surface, which is the interface between the wave and the source, regardless of the values of incident angles [16].

Several techniques have been studied by researchers in the hope of mitigating these drawbacks. One approach involves the use of multiple patches connected to an array or reducing surface wave that can induce an unstable radiation pattern with unexpected ripples [19]. Another technique used to improve the gain and bandwidth is the use of stacked/multilayer patches and/or air gaps between the patches, but this method increases the overall antenna volume [20,21]. Frequency selective surface (FSS) and/or electromagnetic bandgap (EBG) structures have also been proposed by several researchers for antenna bandwidth and gain improvements [8,22]; however, these techniques increase the overall thickness of the antenna and enhancement in the antenna performance is still needed. The use of partial reflective surfaces (PRS) linked with an artificial magnetic conductor (AMC) has also been proposed as a method of overcoming the thickness problem [9,23]. Although the total thickness is appropriately reduced with this approach, the aperture efficiency remains low. One straightforward way to improve the patch antenna bandwidth and gain is to use a thicker substrate, which increases the fraction of total power excited towards the surface wave. The expelled power resulting from the direct irradiation of the material causes degradation of the radiation pattern and of the efficiency of the antenna. An alternative method was proposed that involved the use of multilayered substrates that increase the surface wave coupling between antenna elements; however, mutual coupling can induce impedance mismatch, radiation loss and polarization distortion [24]. A photonic band gap substrate is also an effective means of improving the antenna bandwidth by suppressing harmonics and is one of the most efficient techniques for achieving antenna bandwidth and gain improvements using a metasurface [25,26]. Several reviews focused on metasurface based antenna performance enhancement cover these topics in detail [14,22,24,25,27,28]. Using a metasurface superstrate (MSS), the antenna bandwidth can experience simultaneous increases in gain and reductions in mutual coupling. The reduction of the mutual surface wave coupling effect leads to enhancements in the operating bandwidth. For such cases, the cavity is not essential for designing compact, low profile structures with high radiation efficiency. 

Considering the aforementioned studies and investigations, a square-shaped uniformly distributed metasurface structure with enhanced performance is presented. The proposed metasurface is composed of small slotted dual band patch antenna superstrates that effectively improve the bandwidth and gain. To achieve the targeted dual band characteristics, three horizontal and three vertical slots were used for the lower band and truncation, while two opposite corner slots etched from the rectangular radiating patch were used for the upper band. The antenna with MSS loading has substantially enhanced bandwidth and gain at the lower and upper bands, respectively, with a minimal increase in the overall size. The proposed metasurface superstrate-loaded antenna shows in-phase reflection of the incident waves, which leads to increases in the antenna performance traits, such as bandwidth, gain and directivity. The sufficient bandwidth, gain and radiation characteristics of the proposed MSS-integrated antenna make it suitable for RFID and WLAN applications.

## 2. Design and Configuration 

**Figure 1:**
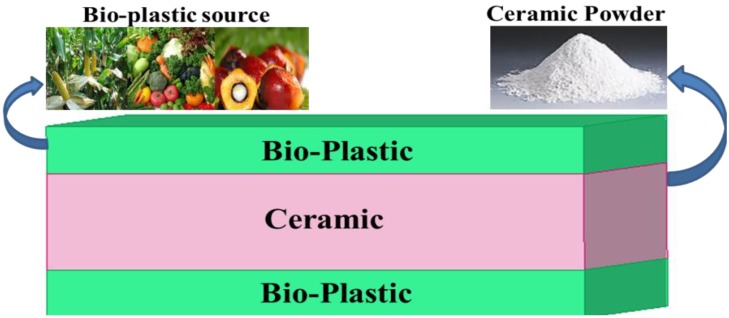
Substrate material structure.

The proposed antenna with a metasurface superstrate structure loading was designed and analyzed using the commercially available finite element method-based high frequency full wave electromagnetic simulator HFSS v.13 from Ansys Corp. The proposed antenna consists of slotted radiating patches on a ceramic-filled bioplastic sandwich-structured substrate with a dielectric constant (*ε_r_*) of 15 [29] and a partial ground plane. Figure 1 shows the proposed sandwich-structured substrate material that was prepared using the bioplastic and ceramic powder. The assorted ceramic powder with a polymeric binder was sintered using the polymeric sponge method, which involves the use of 9.8 mils (0.25) bioplastic sheet from organic biomass sources, such as vegetable oil, cornstarch and palm oil, as the ceramic cover. The three-layered bioplastic-ceramic-bioplastic sandwich structure was laminated with 35 µm of copper foil. The reduced ground plane was used to obtain a wide bandwidth, and the operating bandwidth was further widened using the metasurface superstrate. The detailed design specifications of the radiating patch and MSS are tabulated in Table 1.

**Table 1:** Detail design specification of the proposed antenna.

Parameter	Value (mm)	Parameter	Value (mm)
L1	80	W1	70
L2	2	W2	60
L3	55	W3	2
L4	15	W4	15
L5	15	W5	15
L6	10	W6	2
Lm	8	Wm	8
Lg	2	Wg	2

**Figure 2:**
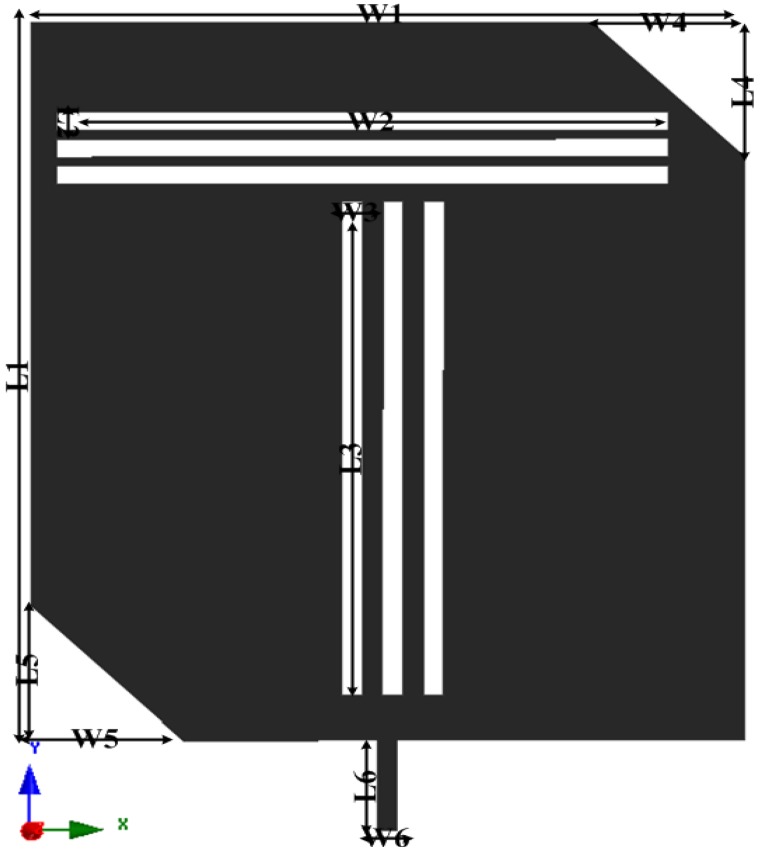
Schematic of the radiating patch.

**Figure 3:**
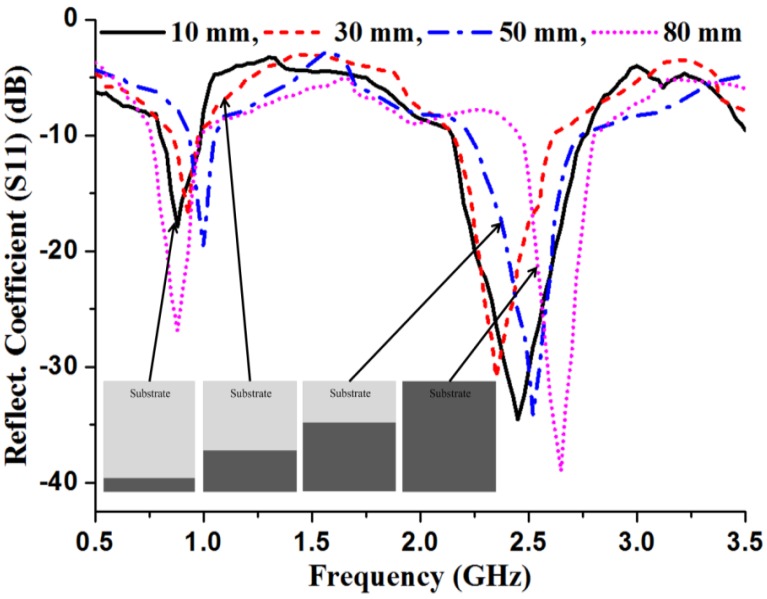
Effect of ground plane length on reflection coefficient of the proposed antenna.

The design process starts with the determination of the overall dimensions of the radiating patch, substrate and ground plane. The basic derivation of the antenna size was adopted from the commonly used mathematical formulation of patch antennas. The available mathematical equations are based on the conventional rectangular patch antenna [30]. However, the length and width of the proposed slotted patch antenna were optimized using the HFSS optimization tool optimetrics. Figure 2 shows a schematic of the radiating patch with optimized dimensions. In an 80 mm long and 70 mm wide (0.2λ × 0.25λ) rectangular patch, three 2 mm × 60 mm horizontal cutting slots and three 55 mm × 2 mm vertical cutting slots are etched to achieve the lower band. To construct the upper band, two opposite corners, measuring 15 mm × 15 mm, are cut out to achieve truncation. Corner truncation, horizontal cutting slots, and vertical cutting slots change the current flow and direction, which allows the antenna to operate in dual band. To reduce the overall size of the antenna, a 2 mm thick high permittivity ceramic-filled plastic substrate with a dielectric constant (*ε_r_*) of 15 was used. The bandwidth of the antenna degrades with the permittivity of the dielectric substrate material [31]. To overcome this bandwidth degradation, the length of the metallic ground plane is reduced to 10 mm; however, this alteration does affect the antenna gain. The effects of ground plane length on the reflection coefficient of the proposed antenna were analyzed, as shown in Figure 3. The optimized 10 mm long ground plane exhibits wider bandwidths for the bands of interest centered at 0.9 GHz and 2.5 GHz. However, the full length (80 mm) of the ground plane lowers the reflection coefficient value while narrowing the bandwidth in the upper band. Thus, there is a tradeoff between antenna size, bandwidth and gain. Therefore, the setup requires an additional element to enhance the antenna performance with minimum dimensions; for these reasons, the metasurface superstrate is placed over the radiating patch without any air gap to enhance the bandwidth and gain.

**Figure 4:**
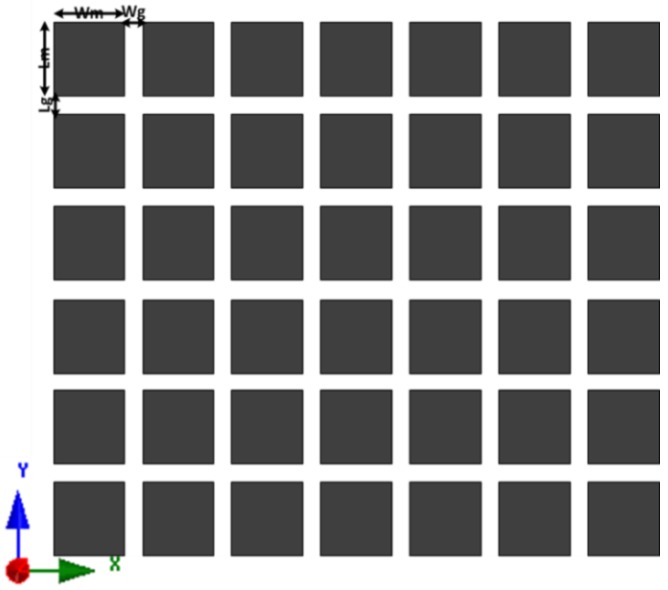
Schematic of the proposed 7 × 6 element metasurface.

**Figure 5:**
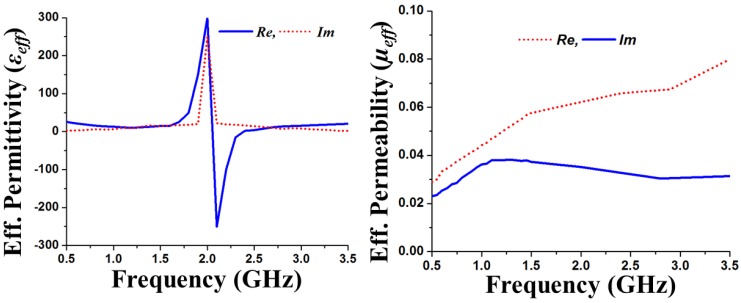
Effective permittivity and effective permeability of the metasurface superstrate structure (MSS) loaded antenna.

**Figure 6:**
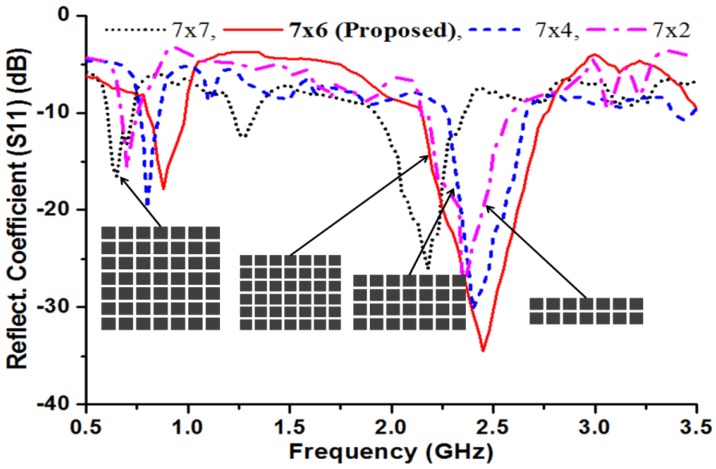
Effect of metasurface element sets over the reflection coefficient of the proposed antenna.

The proposed metasurface superstrate for the antenna is graphically illustrated in Figure 4, and it consists of a planar 7 × 6 matrix with 0.02λ × 0.02λ square-shaped conducting elements with 0.006λ gaps between each element. Square-shaped metasurfaces have been studied by several researchers because of their superior performance [32,33,34]. The dimensions of the MSS-incorporated antenna were optimized using computer-aided finite element method-based optimization tools. A constrained superlinearly convergent active-set algorithm employed the HFSS optimization tool optimetrics to provide the minimized cost function. Consequently, the length and width of the MSS element are optimized. The proposed structure can be defined as a metasurface by identifying the effective parameters. With the aid of commonly used effective parameter retrieval methods [35,36], the effective permeability (*µ_eff_*) and effective permittivity (*ε_eff_*) are obtained, as shown in Figure 5. It is clearly observed that the proposed metasurface exhibits an effective permeability of 0 < *µ_eff_* < 1. With the implementation of commonly used mathematical formulations [37], the corresponding refractive index *n* can be calculated as $n=\sqrt{\varepsilon_{eff}\times \mu_{eff}}$. Therefore, the refractive index *n* also approaches zero because it is proportional to the effective permittivity and effective permeability. The MSS was designed on a 1 mm thick high permittivity dielectric (*ε_r_* = 15) ceramic-filled bioplastic substrate. The effect of the metasurface element matrix on the reflection coefficient was studied and is shown in Figure 6. The optimized 7 × 6 element set offers a better reflection coefficient value and wider bandwidth compared to the other three element sets. 

**Figure 7:**
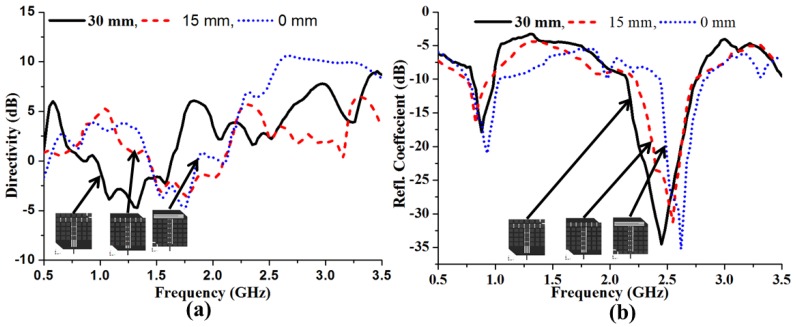
Influence of the MSS position on the directivity (**a**); and (**b**) reflection coefficient of the proposed antenna.

The core concept in designing the metasurface is to stimulate resonant oscillations in the superstrate attachments, which results in a wider radiation aperture. At specific frequencies, the currents induced from the conducting elements negate the incident wave and prevent it from propagating through the set of elements. To broaden the bandwidth in both the upper and lower bands, the optimized 2 mm gap was maintained between elements. In this scenario, two different behaviors can be observed due to the capacitive and surface wave coupling effects. Therefore, it will react to either cancel the current of the elements with each other or to perfectly choose the element size to merge both operating bands together. Principally, the annulment of the total current does not correspond to the elimination of emission given that the superstrate is three-dimensional. Thus, every element in the superstrate will radiate ideally in an analogous way, allowing an unvarying aperture phase circulation. The mutual coupling effect between the elements of the metasurface is a critical point that must be addressed; however, it is diminished by tuning the optimal element size and spacing gap. From the parametric analysis of the MSS starting point, the feed point of the radiating patch is determined. Figure 7 shows the effect of MSS starting position with respect to the feed point over the directivity and reflection coefficient of the proposed antenna. The position of the MSS over the radiating patch has a major influence on the reflection coefficient and directivity. It can be observed that a 30 mm starting point provides the expected resonant frequencies as well as lower and upper bandwidth values. Conversely, the same MSS starting and feed points offer better directivity, but the resonant frequencies are shifted to higher values and the bandwidth is negatively impacted. Therefore, a MSS starting point of 30 mm is chosen to achieve a wide bandwidth and the expected resonant frequencies with acceptable directivity.

**Figure 8:**
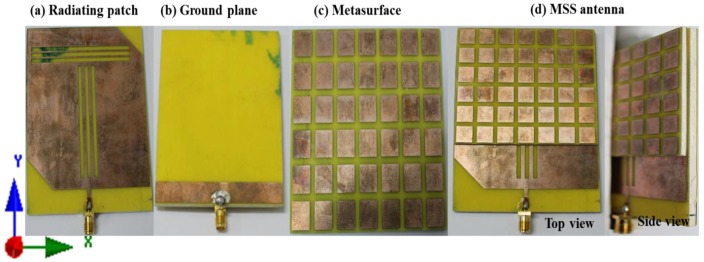
Photograph of fabricated prototype of (**a**) radiating patch; (**b**) ground plane; (**c**) metasurface; and (**d**) metasurafce superstrate loaded antenna.

The proposed slotted patch antenna with a metasurface superstrate was prototyped using a LPKF in-lad PCB prototyping machine for verification of the performance results. Figure 8 shows a photograph of the fabricated prototype (a) radiating patch; (b) ground plane; (c) metasurface superstrate; and (d) proposed metasurface superstrate-loaded antenna. A 10 mm long and 2 mm wide microstrip line is used for feeding to meet the standard 50 Ω impedance characteristics. An SMA connector is used at the end of the microstrip feed line and at the middle of *X* and *Y* axes intercept for excitation.

## 3. Results and Experimental Verification

**Figure 9:**
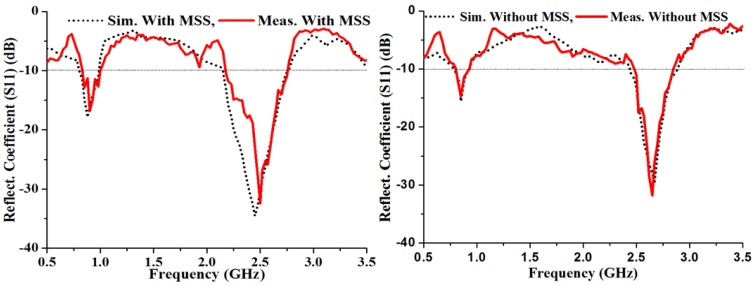
Simulated and measured reflection coefficient of the proposed antenna.

**Figure 10:**
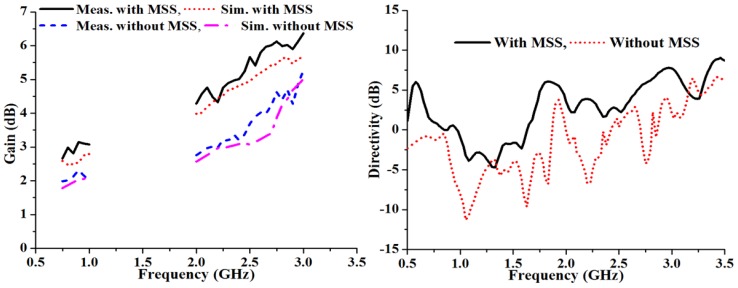
Gain and directivity of the proposed antenna with MSS and without MSS.

To validate the simulated results of the proposed antenna, the performance of the fabricated prototype was measured in a standard 5.5 mm × 5 mm × 3.5 mm anechoic measurement chamber. An Agilent vector network analyzer (VNA) was used for the measurement procedure. The simulated and measured reflection coefficients of the proposed antenna are presented in Figure 9, where it is evident that there is good agreement with and without the metasurface. It is clearly observed from the measured reflection coefficient that the metasurface superstrate-loaded antenna has an increased bandwidth from 120 MHz (0.81–0.93 GHz) to 170 MHz (0.83–1.00 GHz) in the lower band and from 370 MHz (2.48–2.85 GHz) to 580 MHz (2.18–2.76 GHz) in the upper band. The metasurface superstrate loading suppresses the mutual coupling effect, and as a result, the bandwidth is widened. Figure 10 shows the gain and directivity of the proposed antenna, which are corroborated by the simulated results. Additionally, these results reveal that the transmitting wave is more consistent in the lower, rather than the upper, band. The directivity of the proposed antenna increased significantly by including the MSS, and there is good agreement between the gain and directivity of the proposed antenna. The directivity increased in the upper band as the gain was lowered. For the gain measurement, the three antenna measurement system was used with two identical horn antennas. It can be observed that the measured gain was significantly increased with the incorporation of the metasurface superstrate in the lower and upper bands compared to the antenna alone. The average measured gain improved from 2.12 dBi to 3.02 dBi in the lower band and from 4.10 dBi to 5.28 dBi in the upper band.

**Figure 11:**
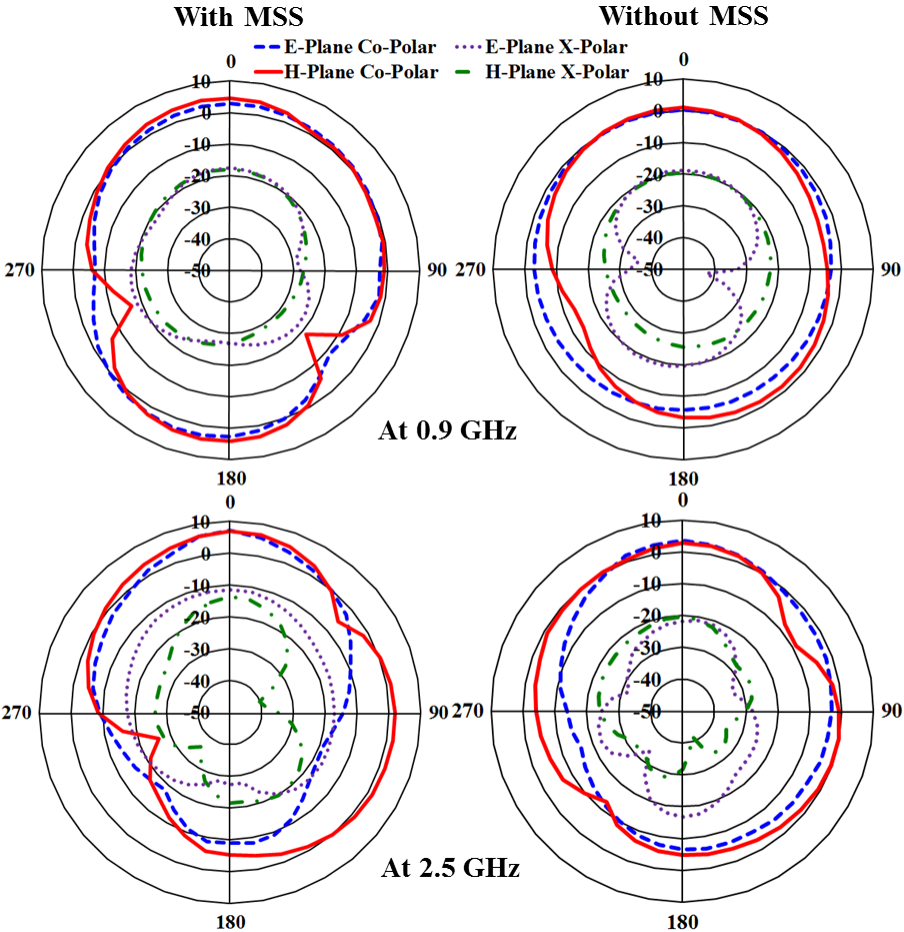
Measured radiation pattern of the antenna at both resonant frequencies with and without MSS.

**Figure 12:**
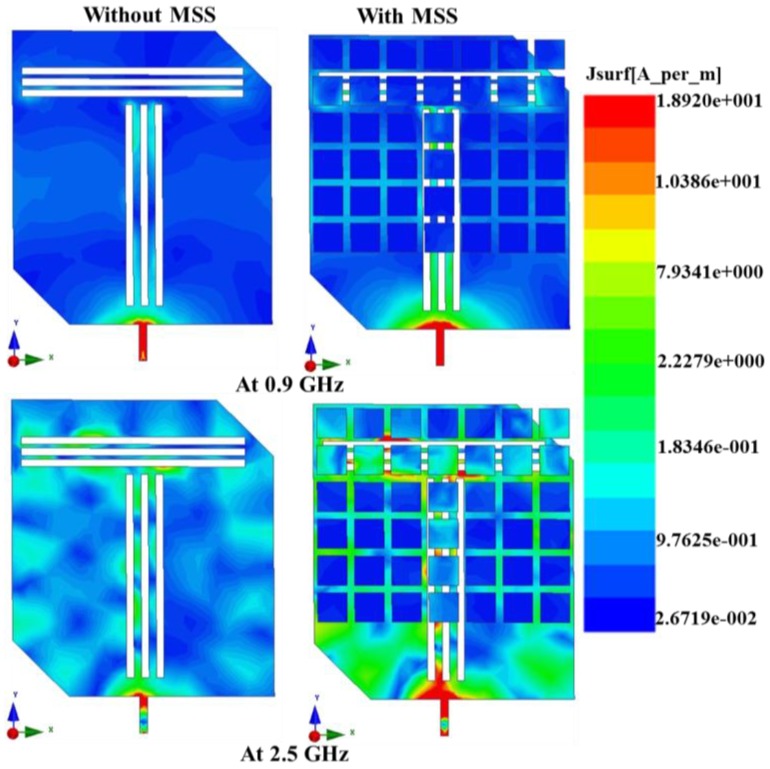
Surface current distribution of the proposed antenna with and without MSS at both lower and upper resonant frequencies.

**Figure 13:**
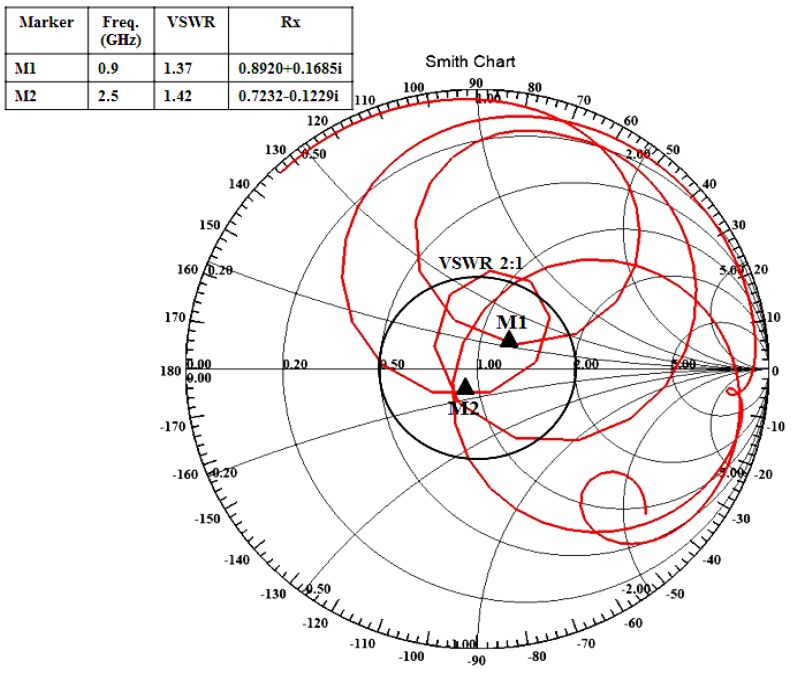
Smith chart of the proposed MSS loaded antenna.

The measured E and H plane radiation patterns for the proposed antenna in both the lower and upper resonant frequencies, 0.9 GHz and 2.5 GHz, respectively, with and without the MSS are shown in Figure 11. In addition to the bandwidth and gain enhancements, the MSS-incorporated antenna exhibits more directional radiation characteristics than that without MSS; these effects are not desired for RFID and WLAN applications. For specific applications such as RFID and WLAN, the radiation pattern is expected to be omnidirectional. However, the radiation pattern of the proposed antenna adopts a directional component with an increase in gain. The broadside beamwidth of the MSS-loaded antenna decreases compared to the patch antenna alone, and as a result, the directivity is increased. Moreover, the backward radiation is slightly higher, but such an effect can be caused by a poor cable connection between the antenna and the controller. The surface current distribution of the proposed antenna is shown in Figure 12. From the surface current distribution, it can be observed that the excited power flow is stronger and consistently in the upper band compared to the lower band. It can also be observed that the antenna with MSS loading exhibits a more intensely distributed current in both the lower and upper bands compared to the patch antenna itself. The input impedance and voltage standing wave ratio (VSWR) of the proposed MSS-integrated patch antenna were validated using the Smith chart in Figure 13. It can be clearly observed that both the lower and upper resonant frequencies are within the VSWR 2:1 circle, which means that the values are less than two. The two resonant frequencies are denoted as M1 and M2, and the VSWR values and input impedance Rx are tabulated in the marker table. The lower resonance in the Smith chart lies above the zero line, and thus shows a positive imaginary value. In contrast, the upper resonance lies below the zero line, which marks it as a negative imaginary value. The detailed performance specifications of the individual proposed MSS antenna and patch antenna are presented in Table 2. 

**Table 2:** Performance specifications of the proposed antenna with and without MSS.

No.	Parameters	Antenna with MSS		Antenna without MSS	
		Lower band	Upper Band	Lower band	Upper Band
1	Overall Dimension	90 mm × 70 mm × 3 mm		90 mm × 70 mm × 2 mm	
2	Bandwidth	18.8%	23.2%	13.3%	14.8%
3	Min. reflection coefficient value	−16.8 dB	32.4 dB	14.7 dB	31.8 dB
4	Max. Gain	3.14 dBi	6.12 dBi	2.15 dBi	4.69 dBi
5	Max. Directivity	0.58 dB	6.20 dB	−0.48 dB	2.89 dB

## 4. Conclusions

A new single-sided metasurface superstrate-loaded dual band patch antenna on a high permittivity ceramic-filled bioplastic sandwich-material substrate was designed and analyzed for bandwidth and gain enhancements. A prototype of the proposed antenna was fabricated and measured for experimental verification. Based on the measurement results, substantial improvements in the bandwidth and gain were realized. The MSS superstrate-loaded antenna widened the operating bandwidth from 120 MHz to 170 MHz and from 370 MHz to 580 MHz for the lower and upper bands, respectively. Moreover, the measured average gain improved from 2.12 dBi to 3.02 dBi in the lower band and from 4.10 dBi to 5.28 dBi in the upper band. In addition to the bandwidth and gain enhancements, the MSS-integrated antenna exhibits a more directional radiation pattern compared to the patch antenna alone. A parametric analysis of the MSS element sets and ground plane length of the patch antenna was conducted. The input impedance and VSWR of the proposed antenna have further validated the use of the Smith chart.
